# The impact of a community social marketing campaign on children’s meal orders and consumption: main outcomes from a group randomised controlled trial

**DOI:** 10.1017/S136898002200163X

**Published:** 2022-08-08

**Authors:** Erin Hennessy, Eleanor Shonkoff, Linda Harelick, Peter Bakun, Kenneth Chui, Susan Roberts, Sara Folta, Jeanne Goldberg, Christina D Economos

**Affiliations:** 1 Gerald J. and Dorothy R. Friedman School of Nutrition Science and Policy, Tufts University, 150 Harrison Avenue, Boston, MA 02111, USA; 2 Merrimack College, North Andover, MA, USA; 3 Harlem Children’s Zone, New York, NY, USA; 4 Tufts University, School of Medicine, Boston, MA, USA; 5 Jean Mayer USDA Human Nutrition Research Center on Aging, Medford, MA, USA

**Keywords:** Dietary intake, Plate waste, Calories ordered, Calories consumed, Social marketing campaign

## Abstract

**Objective:**

Restaurants may be important settings for interventions to reduce children’s energy intake. The objective of this study was to test the impact of a parent-focused social marketing campaign to promote healthy children’s meals on calories ordered and consumed by children at quick-service restaurants (QSR).

**Design:**

Using a repeated cross-sectional study design, two urban communities were randomised to intervention (IN) *v*. control (C) condition. A community-wide social marketing campaign was implemented in the IN community to empower Black and Latinx mothers who frequent QSR (priority population) to select healthier options for their child.

**Setting:**

Data were collected in 2016 at QSR located within the communities pre- and post-IN and analysed in 2017.

**Participants:**

Parents (*n* 1686; *n* 819 and *n* 867 for I and C conditions, respectively) were recruited after placing their QSR order; a survey, receipt and their child’s leftovers were collected.

**Results:**

Calories ordered did not differ significantly between the IN and C conditions (change_adj_ = –146·4 kJ (–35·0 kcal); 95 % CI –428·0 kJ (–102·3 kcal), 134·6 kJ (32·2 kcal)). In a sub-analysis of only the priority audience, children in the IN community ordered significantly fewer calories compared to C children in unadjusted models (change_unadj_ = –510·4 kJ (–122·0 kcal); 95 % CI –1013·4 kJ (–242·2 kcal), –7·5 kJ (–1·8 kcal)), but the trend did not persist after adjusting for covariates (change_adj_ = –437·2 kJ (–104·5 kcal); 95 % CI –925·5 kJ (–221·2 kcal), 50·6 kJ (12·1 kcal)). Calories consumed followed similar trends.

**Conclusion:**

The campaign did not significantly reduce children’s QSR calories ordered or consumed. However, a quantitatively important mean reduction in calories was suggested among the priority audience, indicating potential for community-wide promotion of healthful children’s meals.

Approximately one-third of US children consume food from a quick-service restaurant (QSR) each day, and this consumption has been associated with higher caloric intake^(1–3)^. However, there is limited research evaluating interventions that have targeted individual behaviour change to promote healthy meal consumption with reduced calorie intake in the QSR setting beyond restaurant menu labelling. Social marketing campaigns have demonstrated effectiveness in changing behaviour^(4–6)^, yet, to our knowledge, no campaign has focused on children’s dietary intake in QSR. We designed and tested a social marketing campaign promoting healthy food and beverage choices consistent with national recommendations^(7)^. Given the important role mothers play in shaping children’s eating behaviours^(8,9)^, the campaign goal was to empower mothers to select healthier options when eating from QSR.

## Methods

### Intervention description

**Fig. 1:**
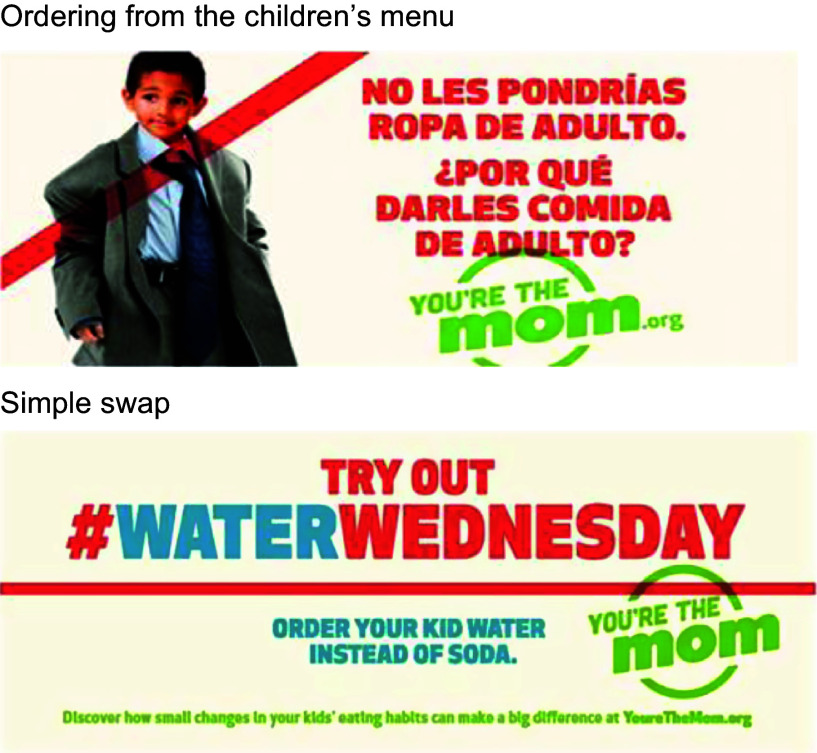
You’re the Mom social marketing campaign execution examples

Details on the development and nature of the campaign are the subject of a separate paper but are summarised briefly here. Using a social marketing framework, the campaign was designed through extensive formative research (e.g. ideation sessions, copy tests, focus groups, etc.), which indicated among other things that mothers found the concept of ‘right-sizing’ a child’s QSR meal highly motivating and fun and wanted more information about achievable, easy strategies they could employ to select healthy options when they visit QSR^(7)^. An advertising agency then developed the creative strategy and executional elements for the *You’re the Mom* campaign (https://yourethemom.org/) for Black and Latinx mothers who frequent QSR (defined prospectively as the priority population). The underlying assumption was that developing messages around an empowerment theme would resonate and inspire the priority audience given that they often do not experience high levels of agency, choice or autonomy in their lives due to financial constraints, discrimination and other factors^(10)^. Utilising an empowerment theme, our campaign reminds mothers that they do have a choice when it comes to ordering for their children in QSR. Figure 1 illustrates two campaign executions. Messages focused on ordering from a children’s menu and making simple swaps (e.g. ordering milk/water *v*. soda; messaging and imagery was not specific to the QSR restaurant) illustrating how making healthy food choices can be easy and fun. The campaign ran for 16 weeks (June–September 2016). Guided by a community advisory board, dissemination occurred through billboards, public bus interior posters, live radio, social and digital media, outdoor murals, painted utility boxes, flyers, banners and community events.

### Setting/study population

Two large (over 100 000 people) urban communities in Massachusetts were selected based on demographic similarity and the presence of multiple outlets of the same QSR. Both communities were considered low income (% families with children over the age of 18 years in the household living in poverty *v*. state average of 13 %)^(11)^. One community was randomised to receive the (IN)tervention (∼40 % families living in poverty) and the other to the (C)ontrol (∼30 % families living in poverty) condition. Overall, the population of the IN community was approximately 25 % Black and over 40 % Hispanic while the population in the C community was approximately 15 % Black and 20 % Hispanic.

The randomisation was carried out using software R’s ‘sample’ function. To ensure the result was open and repeatable, a random seed (which was the sum of numbers ranging from 0 to 999, individually provided by the eleven study personnel) was set prior to the random allocation. After the randomisation, the final result, as well as the numbers collected, was announced together with the software code.

To facilitate evaluation and following a repeated cross-sectional study design, eleven QSR locations from the partnering chain participated (65 % of total locations): six in the IN community and five in the C community. To assess eligibility and interest, research assistants approached parent–child dyads in the QSR *after* orders had been placed. Eligibility criteria included (a) parent/legal guardian over aged 18 years with at least one child aged 4–12 years present; (b) purchase of food/beverage; (c) parent lived, worked or frequently travelled to the community (IN community only) and (d) for post-measurement only (T2): did not participate at baseline (T1).

Eligible participants selected one of two conditions: (a) complete a survey and provide receipt or (b) complete a survey, provide receipt *and* provide child’s leftover food/beverage. Participants received a $10 gift card. Cross-sectional data were collected 8 weeks before the campaign (T1) and the last 4 weeks of the campaign plus 4 weeks post-campaign (T2). Data collection occurred around lunch and dinner on two weeknights and one weekend day. Research activities were conducted in English and Spanish. Tufts University Institutional Review Board approved this study.

#### Power analysis

Sample size was first estimated using a cross-sectional setting due to the lack of pre-existing comparable serial cross-sectional data. The required sample was then doubled as a conservative sampling target. To test the hypothesis that a campaign could reduce calories ordered by parents and consumed by children, two major outcomes, calories ordered and calories consumed, were selected. Based on our previous work, the mean ± sd of calories ordered and consumed were 3205 kJ ± 1255 kJ (766 kcals ± 300 kcals) and 3067 kJ ± 1121 kJ (733 kcal ± 268 kcal); site-level intraclass correlation coefficient was ∼0·007. With resource optimised, we expected to recruit 1200 participants from twelve sites at each time point, yielding a design effect of 1·69 (1 + (1200/12 – 1) × 0·007), and thus an effective sample size of 708 after adjusting for clustering. Using this number and the descriptive statistics, we conducted Markov Chain Monte Carlo simulations to simulate two-time-point scenarios in order to estimate the detectable difference-in-differences. Type I error rate was set at 5 %. According to the results, for calories ordered, we have 80·6 % power to detect a difference-in-difference greater than 377 kJ (90 kcal). For calories consumed, our pilot study showed that ∼40 % of respondents contributed plate waste data, so our effective sample size was decreased to 377 per time point (design effect revised to 1·27). Thus, we would have 81·0 % power to detect a difference-in-difference greater than 460 kJ (110 kcal).

### Measures

Using receipts, research assistants recorded items ordered for the child and asked the parent whether any items were shared. Item codes from the receipt were linked to the QSR’s Nutrient Information System. Methodologies developed and validated in our laboratory were used to estimate calories consumed^(12–14)^. In brief, weight in grams of complete servings (‘pre-consumption portion’) was provided by the QSR. Post-meal, participants provided the child’s leftover food and beverage. Each leftover item was weighed to the nearest gram twice in complete packaging using a digital scale (OXO 1130800, OXO Company). Liquids (e.g. beverages/melted ice cream) were measured in fluid ounces using research-grade containers. Percentage consumed was calculated for each item using the following:

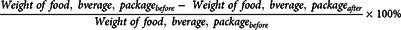




Calories per gram were calculated based on the restaurant’s Nutrient Information System data. Percentage consumed was multiplied by calories per gram to determine calories consumed for each item. All items were summed for total calories consumed during the meal.

Parents reported age, race/ethnicity and highest education level attained as well as frequency of QSR consumption.

### Statistical analysis

Descriptive statistics were tabulated. A linear mixed model was used to account for QSR-level clustering examining calories ordered for and consumed by children. The main variable of interest was condition assignment*time point interaction to measure the effect across time. Covariates were child’s age, sex, race/ethnicity, parental education level and QSR visit frequency for the full sample. For the priority sample, covariates include child’s age and sex. Race/ethnicity was included as binary (Black/Hispanic), and education level and visit frequency were dropped due to being reduced to a constant. Statistical significance was declared if *P*-value was < 0·05. Stata 14 was used for data management and analysis. The main emphasis of the campaign was directed towards children aged 5–10 years, so models were run and are presented for all mothers with children in that range (full sample) and mothers with children 5–10 years who reported their race/ethnicity as Black or Latinx and frequented QSR more than twice a month (priority audience).

## Results

**Fig. 2:**
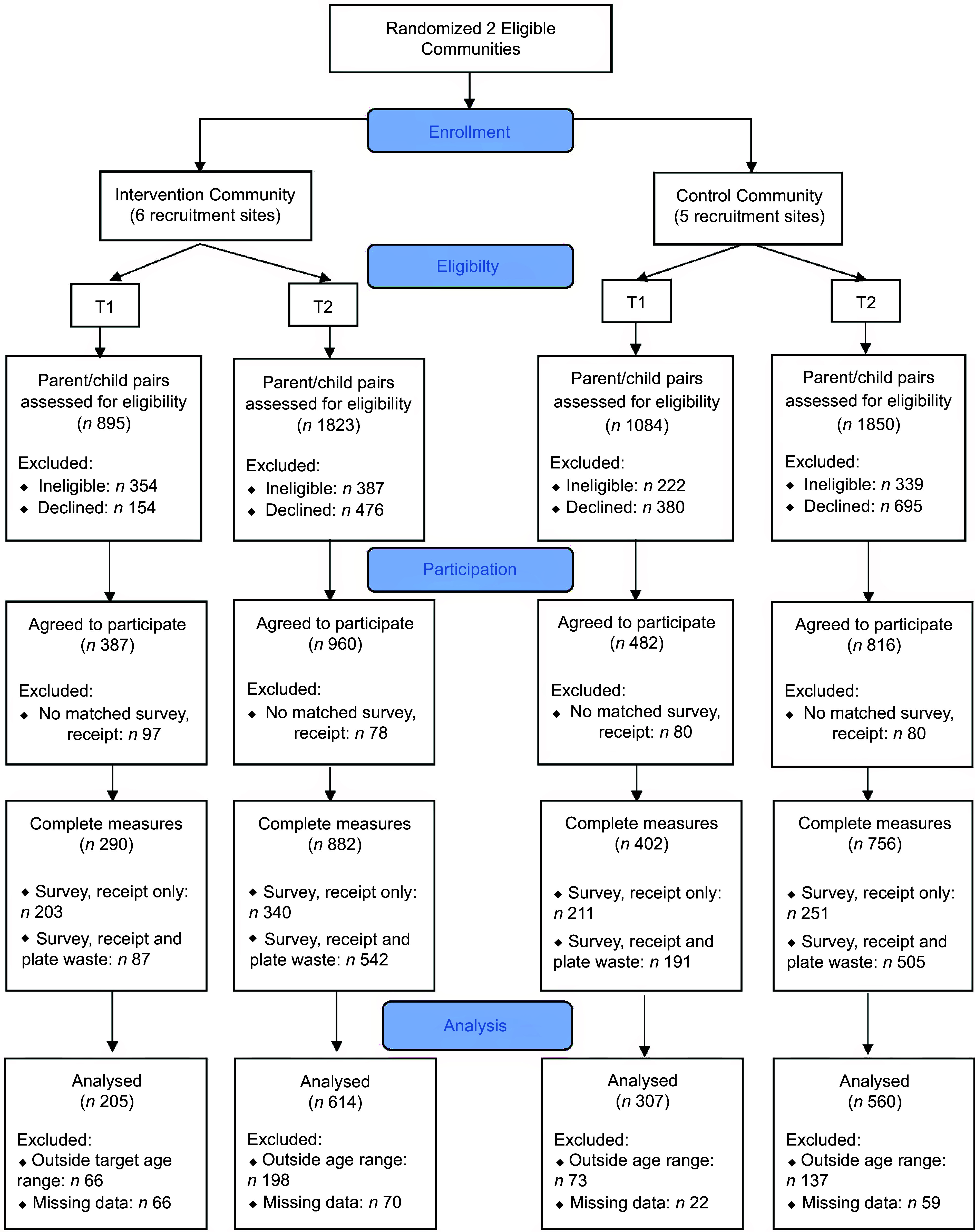
CONSORT flow diagram for the You’re the Mom community-wide social marketing campaign intervention trial. T1, timepoint 1: pre-I baseline; T2, timepoint 2: post-I follow-up

Table 1 and Fig. 2 show demographic characteristics and study participation rates. Mean calories ordered and consumed at each time point in each condition are illustrated in Table 1. There was no statistically significant difference between calories ordered for the IN and C condition in the unadjusted (Change in control – change in intervention = –193·7 kJ (–46·3 kcal); 95 % CI –485·8 kJ (–116·1 kcal), 98·3 kJ (23·5 kcal)) or adjusted models (–146·4 kJ (–35·0 kcal); 95 % CI –428·0 kJ (–102·3 kcal), 134·6 kJ (32·2 kcal)) for the full sample. Among the priority audience, compared with controls, children in the intervention community showed a statistically significant favourable mean difference: (–510·4 kJ (–122·0 kcal); 95 % CI –1013·4 kJ (–242·2 kcal), –7·5 kJ (–1·8 kcal)), but the trend did not persist after adjusting for covariates (mean diff = –437·2 kJ (–104·5 kcal); 95 % CI –925·5 kJ (–221·2 kcal), 50·6 kJ (12·1 kcal)).

**Table 1:** Descriptive characteristics (5–10-year-old sample), *n* 1686*

	Intervention				Control			
	T1 (*n* 205)		T2 (*n* 614)		T1 (*n* 307)		T2 (*n* 560)	
	Mean	sd	Mean	sd	Mean	sd	Mean	sd
Child								
Age	7·40	1·71	7·11	1·65	7·20	1·65	7·17	1·75

	%		%		%		%	
Sex, female	48·29		48·05		50·49		49·46	
Race/ethnicity								
Non-Hispanic White	13·66		11·89		28·99		38·39	
Non-Hispanic Black	10·24		13·19		10·42		8·21	
Hispanic	72·68		66·94		53·09		39·29	
Other	0·98		2·61		5·86		6·79	
N/A	2·44		5·37		1·63		8·21	

	Mean	sd	Mean	sd	Mean	sd	Mean	sd
Calories								
Ordered	714·4	319·7	700·4	309·9	733·1	317·5	760·5	357·2
Consumed	563·6	238·8	544·9	233·2	568·3	274·2	536·3	240·5
Parent								
Age	35·65	8·92	35·00	8·76	36·09	8·43	37·03	8·25

	%		%		%		%	
Sex, female	75·74		73·82		76·62		71·66	
Education level								
≥ Bachelor’s degree	15·12		12·54		21·50		28·04	
Frequency of QSR consumption								
≥ 2–3×/month	70·73		72·80		66·78		61·43	

QSR, quick-service restaurant.

* In the regression models, the sample size for calories ordered in the full sample was *n* 1686 and among the priority audience, *n* 518. The sample size for calories consumed for the full sample was *n* 958 and for the priority audience, *n* 286.

In the full sample, analyses of calories consumed indicated that the change showed a higher mean difference, albeit non-significant, among children in the IN community in unadjusted (Change in control – change in intervention = 117·6 kJ (28·1 kcal); 95 % CI –217·6 kJ (–52·0 kcal), 451·9 kJ (108·0 kcal)) and adjusted models (117·2 kJ (28·0 kcals); 95 % CI –191·8 kJ (–45·84 kcal), 425·5 kJ (101·7 kcal)). In the priority audience, the difference-in-difference models demonstrated a non-significant reduction of 106·3 kJ (25·4 kcals) (95 % CI –487·9 kJ (–116·6 kcal), 484·9 kJ (115·9 kcal)) and 22·2 kJ (5·3 kcal) (95 % CI –565·3 kJ (–135·1 kcal), 530·5 kJ (124·4 kcal)) in the unadjusted and adjusted models, accordingly.

## Discussion

The *You’re the Mom* campaign utilised a unique approach to reach an important audience. While it did not significantly reduce calories ordered for or consumed by children in the intervention community, there was a non-significant trend towards reduction in calories ordered among the priority audience. The campaign emphasised motivational and positive message framing to empower parents to select healthier menu items for their children in QSR. While the campaign and other social marketing interventions may be a promising approach to shifting children’s consumption at QSR, *You’re the Mom* fell short of producing a statistically significant reduction in calories.

It is unclear whether the lack of a statistical effect is due to the study’s inability to reach the desired sample at baseline (T1). Additionally, given that eleven QSR participated instead of twelve, the detectable difference was slightly higher than our initial power analysis (397 kJ (95 kcal) for ordering and 485 kJ (116 kcal) for consumption). Collecting data for this type of study was time and labour intensive, and despite screening almost 2000 dyads at T1, participation at T1 fell below the target. We were able to achieve a larger sample size at T2 due to hiring and training a larger workforce. Other design features may also be required to achieve significant change such as a longer campaign, a more multifaceted campaign including one with intervention elements at the point of purchase or more diverse messages beyond those tested in the study. Future research could explore these adaptations. Moreover, social marketing campaign interventions focused on priority populations such as those described in this study may also need to be supported by changes to the retail food environment to achieve significance^(15,16)^.

Promoting healthful children’s meal selection at QSR is a critical pathway to improve dietary intake. This is the first study targeting specific parenting practices at QSR using a social marketing campaign. Moreover, it is one of the first studies assessing calories ordered *and* consumed within QSR using rigorous nutrition methodology. Previous work indicates that community-wide social marketing campaigns could achieve population-level change^(4–6,17,18)^, yet little is known about efficacy of changing consumer restaurant behaviour. Results shed light on the feasibility of implementing a social marketing campaign targeted to mothers for influencing child food orders and consumption at QSR.
